# Croup, Bœnninghausen and McNeil

**Published:** 1889-05

**Authors:** P. P. Wells


### CROUP, BŒNNINGHAUSEN AND McNEIL.

EDITORS OF HOMŒOPATHIC PHYSICIAN.

GENTS:—In your issue for March, 1889, your highly esteemed correspondent, whose name is given above, enters the field against the treatment of croup practiced by Bœnninghausen for many years, and with an unparalleled success, and by myself and colleagues for a shorter time, and, so far as I know, to this time, with no one failure to cure, and gives the following three reasons for his disapproval of the method:

1. "It is treating a disease for a name, and not patients, thereby entirely ignoring the treatment of the totality of the symptoms."

There is a seeming foundation for this objection in the fundamental principle which requires the treatment of the sick person and not of any name which fancy or *quasi science* may have given to the sickness to be treated. This principle has not been more clearly recognized and obeyed by any man than it was by Bœnninghausen, and yet he had treated "more than four hundred cases of membranous croup without a loss," when he told me how he had done it. We do not readily see how a better record could have been made by a different treatment. With myself the same treatment has been uniformly successful to this day.

Then, the above principle being admitted as binding in all clinical efforts under the guidance of the law of similars, and the above successes being *facts*, the question asks itself whether here the violation of the principle is not rather seeming than real. The question may be answered in this way perhaps. In our interchange of ideas of sicknesses, we are compelled to use *names* of so-called diseases, and there is no failure of duty to therapeutic law or to its cardinal principles when we so use them. There are names of sicknesses which, when spoken, present to the mind a picture which is so perfectly repeated in the successive examples of this sickness that the name contains in it, to the intelligent mind, a more or less complete expression of the totality of the phenomena of that sickness. No doubt Bœnninghausen's treatment was the outcome of the *facts* of croup, and not in the least of its name.

The Doctor says—

2. "It is an alternation of remedies."

This was certainly hastily spoken. Your correspondent is surely sufficiently intelligent to recognize the difference between *succession* and "*alternation*." Bœnninghausen never alternated remedies, and in his method with croup he made no departure from his life habit in this respect.

And, further—

3. "It is not the most successful way of curing croup."

Has your correspondent a more successful record to present us of croup treated in some other "way." Between four and five hundred cases without a loss is certainly a remarkably good record, and this was given to me by Bœnninghausen himself in April, 1858, as the result of his then past experience with his method. But the Doctor says this "is not the most successful way." What is the better, and where is its record?

P. P. WELLS.

